# Harnessing the Microbiome: CRISPR-Based Gene Editing and Antimicrobial Peptides in Combating Antibiotic Resistance and Cancer

**DOI:** 10.1007/s12602-025-10573-8

**Published:** 2025-05-16

**Authors:** Radwa A. Amen, Yaser M. Hassan, Rawan A. Essmat, Rana H. Ahmed, Marwan M. Azab, Nadia R. Shehata, Mariam M. Elgazzar, Wael M. El-Sayed

**Affiliations:** 1 Department of Biotechnology, Faculty of Science, Cairo University, Cairo, Egypt; 2 Biotechnology Program, Faculty of Science, Ain Shams University, Abbassia, Cairo, 11566 Egypt; 3 Faculty of Pharmacy, Modern University for Information and Technology, Cairo, 11728 Egypt; 4 Biotechnology Program, Faculty of Science, Mansoura University, Mansoura, 35516 Egypt; 5 Molecular Biotechnology Program, Faculty of Science, Helwan University, Ain Helwan, Cairo, Egypt; 6 College of Biotechnology, Misr University for Science and Technology, Giza, 12596 Egypt; 7 College of Biotechnology, Badr University in Cairo, Cairo, Egypt; 8 Department of Zoology, Faculty of Science, Ain Shams University, Abbassia 11566, Cairo, Egypt

**Keywords:** Cancer immunotherapy, CRISPR-Cas9, Metagenomics, Microbiome engineering, Multidrug-resistant pathogens, Personalized therapeutics, Probiotics

## Abstract

The growing crisis of antibiotic resistance and the increasing incidence of cancer have prompted the exploration of innovative approaches, such as gene editing and antimicrobial peptides (AMPs). The human microbiome is integral to various aspects of health, disease, and therapeutic development, influencing metabolic pathways, immune function, and pathogen resistance. Recent advances in gene editing technologies, particularly CRISPR (clustered regularly interspaced short palindromic repeats), have opened new avenues for leveraging the microbiome to address complex medical challenges, including combating multidrug-resistant pathogens and cancer. The microbiome plays a crucial role in combating antibiotic resistance by modulating microbial communities, influencing pathogen survival and susceptibility to treatments. This review explores the microbiome’s dynamic role in metabolic regulation, its contribution to cancer management, and how AMPs help maintain homeostasis and exhibit emerging anticancer properties, supported by both preclinical findings and clinical evidence. Additionally, CRISPR-based microbiome engineering offers potential to enhance host-microbiome interactions, optimizing therapeutic outcomes. The integration of microbiome metagenomics and proteomics has led to the discovery of novel AMPs with targeted anticancer effects. Innovative strategies, such as engineered probiotics and CRISPR-based microbiome engineering, present exciting prospects for next-generation therapies. Despite these advances, the translation of microbiome-based therapies into clinical settings remains challenging due to ethical, regulatory, and ecological hurdles. This review underscores the transformative potential of microbiome-based interventions, emphasizing the role of personalized medicine in maximizing therapeutic efficacy. Furthermore, we also address critical research gaps, limitations, and future directions, including optimizing AMP stability, delivery, and bioavailability, as well as overcoming the regulatory and ethical challenges in clinical translation.

## Introduction

The human microbiome, often referred to as “the hidden organ,” represents a vast ecosystem composed of microorganisms, including archaea, viruses, bacteria, and fungi, that coexist across various body sites such as the skin, gut, lungs, reproductive system, and oral cavity [1–3]. Remarkably, the microbiome’s genetic information is estimated to be over 150 times more abundant than the human genome [4]. The microbiome plays a crucial role in immune regulation and maintaining homeostasis, with the gut microbiota being particularly vital. It supports health by fermenting food, defending against pathogens, stimulating immune responses, and synthesizing vitamins [3, 4]. Homeostasis refers to the stable state of internal conditions maintained by the microbiome, which is crucial for immune regulation and overall health. Additionally, the gut microbiota influences mental health through the gut-brain axis, affecting cognition, behavior, and social interactions in animal models. Similarly, the skin microbiota helps defend against pathogens and plays a role in immune system development, producing AMPs such as β-defensins and cathelicidins [5–7].

AMPs are small peptides that serve as a primary defense against pathogens and exhibit antimicrobial and anticancer properties [8]. AMPs selectively target cancer cells by binding to phosphatidylserine (PS) on their outer membranes, triggering apoptosis while sparing normal cells [9]. Certain AMPs, such as LTX-315 and LL-37, enhance immune responses and induce apoptosis by interacting with the tumor microenvironment (TME) [10, 11]. LL-37 is a 37-amino acid antimicrobial peptide derived from the human cathelicidin antimicrobial peptide (hCAP-18). Its active C-terminal sequence is responsible for its potent antimicrobial, immunomodulatory, and wound-healing properties. LTX-315 is a synthetic antimicrobial peptide derived from human lactoferrin, designed for enhanced potency. It disrupts microbial membranes and stimulates immune responses, making it a promising candidate for cancer immunotherapy and infection treatment, particularly in promoting anti-tumor immune responses. AMPs are generally safer than traditional chemotherapy agents due to their lower toxicity to normal tissues, and their anticancer efficacy can be enhanced through chemical modifications [10].

Dysbiosis refers to an imbalance within the microbiome, a community of microorganisms that can negatively affect the host’s health. The human body harbors multiple microbiomes, with the gut microbiota playing a crucial role in both human health and disease. Dysbiosis, or microbial imbalance, has been linked to various diseases, including cancer, cardiovascular conditions, and neurodegenerative disorders. This imbalance typically occurs when beneficial microbes are lost and pathogenic bacteria proliferate. It can disrupt immune signaling, promote chronic inflammation, and alter metabolic processes, contributing to disease development, including cancer and chronic inflammation [6, 12, 13]. As our understanding of the microbiome’s influence on health expands, gene editing technologies such as CRISPR have emerged as powerful tools to manipulate the microbiome for therapeutic purposes [13]. CRISPR-based gene editing enables precise microbial genome modulation, offering novel strategies for antimicrobial and anticancer therapies [14].

Advances in artificial intelligence (AI) offer new promise for studying the complex dynamics of the gut microbiota. Techniques such as deep learning and machine learning have been employed to retrospectively analyze previous studies, uncovering intricate patterns and associations within microbiome data that were once elusive. These AI-driven approaches enhance our understanding of the link between dysbiosis and various diseases, while also paving the way for the development of targeted therapeutics aimed at restoring healthy microbiota [15].

Antibiotic therapy can significantly impact the human symbiotic microbiome, and drug tolerance or persistence often allows a portion of the pathogen population to survive. Moreover, increasing the dose and duration of antibacterial medications may lead to side effects and toxicity, presenting another challenge in achieving pathogen specificity with novel therapies. In response to long-term selection pressure, bacteria have evolved a variety of defense mechanisms, including the restriction-modification system and the clustered regularly interspaced short palindromic repeats (CRISPR)-Cas system. These mechanisms play critical roles in the propagation of AMR genes and phage infections, acting as bacteria’s innate and adaptive immune systems, respectively. The CRISPR-Cas system, in particular, can be harnessed to combat AMR by selectively targeting and deleting antimicrobial resistance genes within the bacterial genome, effectively re-sensitizing antibiotic-resistant bacteria. Given its ability to manipulate genetic material with precision, CRISPR is emerging as a powerful tool in the fight against antimicrobial resistance [16, 17]. This review aims to explore the emerging therapeutic potential of microbiome-based interventions, particularly through gene editing and AMPs, in combating antibiotic resistance and cancer. We discuss how CRISPR-based microbiome engineering and the use of AMPs could be leveraged to address these pressing medical challenges. The review also highlights critical research gaps, future directions, and the challenges of the translation of these therapies into clinical practice.

## Composition and Diversity of the Human Microbiome

The human microbiome is a complex and highly diverse ecosystem of microbes that inhabit various regions of the body, such as the gut, oral cavity, and skin. Recent studies have shown that the microbiome’s composition differs significantly between individuals, and even within the same individual over time, based on various factors, including diet, lifestyle, and environmental exposure [18]. Trillions of microorganisms, including bacteria, archaea, fungi, and viruses, live symbiotically within the human body, with the digestive tract hosting the largest proportion of this microbial population—over 95% of the total. The composition of gut microbiota includes key taxa such as *Bacteroides*, *Firmicutes*, *Actinobacteria*, *Bifidobacterium*, *Eubacterium*, *Clostridium*, *Peptococcus*, *Proteobacteria*, and *Prevotella*, each contributing uniquely to human health [19].

The diversity within the microbiome is not only structural but also functional. For example, the gut microbiota’s functional diversity is pivotal for maintaining the host’s metabolic processes, synthesizing vitamins, fermenting fiber, and modulating the immune system. Furthermore, microbial diversity in the gut correlates with resilience to diseases and a better response to infections [18]. Studies have shown that diet, particularly fiber-rich and fermented foods, plays a significant role in supporting gut microbial diversity, which is associated with better health outcomes. In contrast, a reduction in diversity (a condition known as dysbiosis) has been linked to a wide range of diseases, from gastrointestinal disorders to autoimmune conditions and even neurological diseases.

While the gut microbiota often receives the most attention, the skin microbiome also plays a crucial role in overall health. Similar to the gut, the skin microbiome is highly diverse, with its composition influenced by factors such as moisture, sebaceous content, and skin pH, which vary across multiple body regions [20]. Beneficial bacteria on the skin contribute to maintaining skin health by producing AMPs that protect the skin against harmful microorganisms, help regulate immune responses and maintain skin barrier function [21]. In addition to bacteria, the skin microbiome also includes fungi like *Malassezia* and *Candida*, and viruses, all contributing to the overall diversity and function of the skin ecosystem. Disruption of this delicate balance can lead to conditions like eczema, acne, and psoriasis, underscoring the skin microbiome's pivotal role in maintaining skin health.

The oral microbiome, similarly, is another critical microbial community, interacting with the immune system and influencing processes such as digestion and systemic health. It is home to both beneficial and pathogenic microbes that contribute to oral health and disease, respectively. Pathogens in the oral cavity can lead to conditions such as periodontitis and dental caries, and oral bacteria can also enter the bloodstream, impacting systemic conditions like cardiovascular diseases and diabetes [22].

The diversity across multiple body sites and the interactions between microbiomes are key to maintaining health. These microbiomes are not isolated but interact with each other and the host’s immune system, forming a dynamic, interdependent ecosystem (Table 1). Understanding these interactions is crucial for the development of microbiome-based therapeutic strategies to promote health, prevent disease, and treat conditions such as skin disorders, gastrointestinal diseases, and oral health issues. By recognizing the interconnectedness of microbiomes, researchers and clinicians can develop more targeted, holistic approaches to address these diverse health challenges.

**Table 1:** Composition and diversity of the human microbiome

Location	Findings	References
Gut microbiome	- Trillions of microorganisms (bacteria, fungi, viruses, archaea) reside in the gut, making up > 95% of the total microbial population - Dominant phyla: *Bacteroides*, *Actinobacteria*, *Bifidobacterium*, *Firmicutes*, *Eubacterium*, *Clostridium*, *Peptococcus*, *Proteobacteria*, and *ProveTella*	[18, 19]
Skin microbiome	- Commensal microbiomes maintain skin barrier function and homeostasis - Composition varies based on skin physiology (moisture, dryness, sebaceous content)	[20, 21]
Oral microbiome	- Highly diverse, including bacteria, microeukaryotes, archaea, and viruses - Plays a role in oral and systemic health by interacting with the host immune system	[22]

### Microbiome Functions

The human microbiome plays a crucial role in immune system development and function. The coevolution of indigenous microbiota with the host immune system has allowed the immune system to distinguish between beneficial commensals, which must be preserved, and harmful pathogens that need to be eliminated. This interplay enhances both innate and acquired immunity. The composition of the gut microbiota significantly shapes the developmental characteristics of the acquired immune system, influencing processes such as immune cell differentiation and antibody production. Studies have demonstrated that gut microbes modulate the production of immune molecules such as cytokines and immunoglobulins, which are essential for defense against infections [23]. Disruption of this balance, known as dysbiosis, is linked to autoimmune diseases, chronic inflammatory conditions, and a compromised immune response to infections.

Beyond immune function, the microbiome plays a key role in nutrition. The colonic microbiota, for example, aids in the digestion of complex, indigestible dietary components such as dietary fiber and polysaccharides. Through fermentation, it produces short-chain fatty acids (SCFAs) like butyrate, propionate, and acetate, which not only serve as energy sources for colon cells but also have anti-inflammatory effects that contribute to gut homeostasis. These SCFAs can also enter the bloodstream and influence systemic health, with effects on metabolism, immune regulation, and even the brain [24]. Additionally, certain bacteria, such as *Bacteroides* spp., *Bifidobacterium* spp., and *Enterobacteria*, are involved in synthesizing essential vitamins, including B vitamins and vitamin K, which are critical for various physiological processes such as blood coagulation and neural function [23].

Commensal bacteria also contribute to host defense by producing antimicrobial peptides and bacteriocins, which specifically target pathogenic bacteria, limiting their colonization. Bacteriocins, which share characteristics with traditional antibiotics, offer a promising alternative or complementary approach to combating harmful microorganisms. Unlike conventional antibiotics, which often have broad-spectrum activity, bacteriocins are more targeted, reducing the likelihood of disrupting beneficial microbiota. Additionally, they have been shown to act synergistically with antibiotics, enhancing their efficacy and overcoming challenges posed by multidrug-resistant pathogens [24]. The discovery and development of bacteriocins as therapeutic agents hold great promise for addressing the growing issue of antibiotic resistance.

The microbiome’s contribution to immune regulation, nutritional support, and antimicrobial defense underscores its integral role in maintaining overall health (Table 2). By supporting immune function, enhancing nutrient absorption, and providing a first line of defense against pathogens, the microbiome is a key player in protecting the body from disease and promoting homeostasis.

**Table 2:** Functions of the human microbiome

Function	Findings	References
Immune system development	- Indigenous microbiotas coevolve with the immune system, aiding in distinguishing pathogens from commensals - Gut microbiota shapes the adaptive immune system and manages microorganisms	[23]
Nutritional contributions	- Colonic microbiotas break down complex dietary constituents, aiding in nutrient absorption and vitamin production (e.g., *Bifidobacterium* spp., *Bacteroides* spp., enterobacteria)	[23]
Antimicrobial production	- Commensal bacteria produce bacteriocins, which inhibit specific pathogens and resemble traditional antibiotics	[24]

### Microbiome-Related Disorders

Dysbiosis refers to an imbalance in the composition and function of the microbiota, particularly the gut microbiota. This disruption can lead to altered mucosal immunity, such as the excessive production of aberrantly glycosylated immunoglobulin A (IgA), which may deposit in tissues like the kidneys, triggering inflammation and progressive organ damage. For example, in the case of chronic kidney disease, this immune dysregulation can have long-lasting health consequences [25]. Beyond the gut, dysbiosis has also been implicated in several systemic diseases, highlighting its widespread impact on human health. Dysbiosis has been closely linked to metabolic diseases such as type 2 diabetes. Machine learning techniques have been instrumental in identifying these relationships by analyzing data derived from the microbiome. For example, random forest models have been used to classify patients as normoglycemic, prediabetic, or diabetic based on their blood glucose levels and gut microbiota profiles obtained through 16S rRNA gene sequencing. These models successfully distinguish between the gut microbiomes of individuals with type 2 diabetes, prediabetes, and those with normal blood glucose levels, while also identifying bacterial species associated with the disease. The findings suggest that dysbiosis may contribute to the development of diabetes, and that modulation of gut microbiota could offer a viable therapeutic approach [15].

Inflammatory bowel disease (IBD), which includes Crohn’s disease and ulcerative colitis (UC), is a classic example of a disorder driven by microbiome dysregulation. Both forms of IBD are marked by chronic inflammation in the gastrointestinal tract, and disruptions in the gut microbiota contribute to this inflammatory response [26]. The microbiome plays a critical role in maintaining intestinal mucosal integrity and modulating immune responses. In IBD, altered microbiota composition can lead to an inappropriate immune response, driving the chronic inflammation seen in these diseases [27].

The growing recognition of the gut-brain axis has led to a surge of interest in the microbiome’s role in mental health, particularly in conditions like major depressive disorder (Table 3). The gut microbiota influences the synthesis of key neurotransmitters, such as serotonin, which are essential for regulating mood and mental well-being [28]. Dysbiosis can result in a decrease in beneficial metabolites, exacerbating depression and anxiety symptoms, and suggesting that modulation of the microbiome may offer new therapeutic avenues for these conditions [29].

**Table 3:** Microbiome-related disorders

Disorder	Key findings	References
Dysbiosis	- Imbalance in gut microbiota composition leads to mucosal immunity alterations, aberrant IgA production, and chronic kidney disease	[19, 25]
Inflammatory bowel disease (IBD)	- Includes Crohn’s disease and ulcerative colitis, mediated by immune system dysfunction and gut microbiota alterations	[26, 27]
Major depressive disorder	- Gut-brain axis links gut microbiota to neurotransmitter synthesis (e.g., serotonin), immune responses, and brain structure	[28, 29]
Antibiotic resistance	- Misuse of antibiotics increases susceptibility to pathogens like *Clostridioides difficile* and contributes to dysbiosis	[30]
Gastric cancer	- Dysbiosis in *Fusobacterium* and *Clostridium* which are overrepresented in this type of cancer	[23, 31]
Colorectal cancer (CRC)	- This imbalance has been linked to an increase in bacteria such as *Fusobacterium nucleatum* and certain strains of *E. coli* in CRC patients. These bacteria can induce local inflammation, produce genotoxins, and alter metabolic profiles, all of which contribute to tumor initiation and progression, a process associated with gut microbiome dysbiosis	[32]
Liver cancer	- Gut dysbiosis leads to disruption of the intestinal barrier, allowing bacterial endotoxins to reach the liver, which exacerbates the inflammatory process and fat accumulation in the pathogenesis of non-alcoholic fatty liver disease	[33]
Breast cancer	- Its development has been associated with a decreased proportion of bacteria from the Bacteroidaceae family and an increased proportion of bacteria from the *Agrococcus* genus. Additionally, microbial profiles vary throughout the progression of breast cancer	[34]
Alzheimer’s disease (AD)	- It occurs as a result of gut microbiome dysbiosis, which leads to alterations in at least five different pathogenic processes. These include amyloid-beta deposition, increased tau phosphorylation, neuroinflammation, metabolic dysfunction, and oxidative stress	[35]
Parkinson’s disease (PD)	- Gut microbiome imbalances may promote alpha-synuclein aggregation, a hallmark of PD. Increased levels of trimethylamine-N-oxide have been linked to PD progression	[36]
Rheumatoid arthritis (RA)	- Gut microbiota alterations may influence immune system activation and inflammation in RA, with patients exhibiting a marked reduction in beneficial bacteria such as *Bacteroides fragilis* and *Roseburia faecis* compared to healthy individuals	[36, 37]
Huntington’s disease	- This occurs due to microbial diversity and altered abundance of specific bacterial taxa, including an increase in Bacteroidetes and a decrease in Firmicutes phyla. These changes lead to increased intestinal permeability, inflammation, and motor deficits in HD mice	[38]

Antibiotic resistance is another critical issue arising from microbiome disturbances. The overuse and misuse of antibiotics disrupt the balance of the gut microbiota, leading to increased susceptibility to infections, including those caused by *Clostridioides difficile* [30]. Antibiotic-induced dysbiosis has also been linked to the increased risk of colorectal cancer, as chronic alterations in microbial composition can influence inflammation and immune responses that promote tumorigenesis. Similarly, microbial imbalances, such as an overrepresentation of *Fusobacterium* and *Clostridium*, have been identified as contributing factors in the development of gastric cancer, paralleling the role of *Helicobacter pylori* in gastric cancer [23, 31].

The expanding body of evidence linking the microbiome to various diseases has spurred interest in therapeutic strategies aimed at restoring microbiome balance (Table 4). These include fecal microbiota transplantation (FMT), the use of probiotics, and microbiome-targeted drugs. Such interventions may offer potential treatments for conditions ranging from IBD to mental health disorders and even cancer, underscoring the therapeutic potential of microbiome modulation.

**Table 4:** Microbiota and their roles in human health

Microbiota	Role/function	Specific examples/clinical applications	References
*Bacteroides* (gut)	Nutrient breakdown, vitamin production, immune modulation	Clinical studies show *Bacteroides thetaiotaomicron* aids in polysaccharide digestion and reduces inflammation in IBD patients	[19, 23, 39]
*Firmicutes* (gut)	Energy harvest, metabolic regulation	*Clostridium* species within Firmicutes are involved in producing butyrate, which has been shown to improve gut barrier function in metabolic syndrome patients	[19, 40]
*Bifidobacterium* (gut)	Vitamin synthesis, pathogen inhibition, immune support	*Bifidobacterium longum* supplementation has been shown to reduce symptoms of IBS and improve gut health in clinical trials	[23, 41]
Commensal skin microbes	Produce antimicrobial peptides (AMPs), maintain skin barriers, protect against pathogens	*Staphylococcus epidermidis* produces AMPs that inhibit *S. aureus* colonization, reducing the risk of skin infections	[20, 21, 42]
Oral microbiota	Maintain oral and systemic health, interact with host immune system	*Streptococcus salivarius* has been used in probiotics to reduce oral pathogens and prevent dental caries	[22, 43]
*Roseburia faecis*	Contributes to the production of SCFAs such as butyrate, which have anti-inflammatory properties. A decrease in this bacterium may result in lower SCFA levels, thereby reducing anti-inflammatory effects and promoting chronic inflammation	Clinical studies suggest that *Roseburia* spp. are reduced in patients with ulcerative colitis, and their restoration is associated with improved outcomes	[37, 44]
*Fusobacterium nucleatum*	It has been linked to modulating T-cell responses. Although it can exhibit dual roles depending on the context, its lower abundance in RA patients may disrupt normal immune regulation, potentially impairing mucosal immunity and promoting systemic inflammation	*Fusobacterium nucleatum* is associated with colorectal cancer progression, and its detection in tumors is a potential biomarker for disease prognosis	[37]
*Faecalibacterium prausnitzii* (gut)	Produces butyrate, has anti-inflammatory properties, and supports gut barrier function	Reduced levels of *Faecalibacterium prausnitzii* are associated with Crohn’s disease, and its restoration is linked to improved gut health	[45, 46]
*Lactobacillus* (gut)	Produces lactic acid, inhibits pathogen growth, supports gut barrier function, and modulates the immune system	*Lactobacillus rhamnosus* has been shown to reduce the severity of diarrhea in children and improve gut health in clinical trials	[47, 48]
*Akkermansia muciniphila* (gut)	Degrades mucin, maintains gut barrier integrity, and regulates metabolic health	Clinical studies suggest that *Akkermansia muciniphila* is reduced in obese individuals, and its supplementation improves insulin sensitivity and reduces inflammation	[46, 49]
*Prevotella* (gut)	Involved in carbohydrate metabolism and immune modulation	*Prevotella copri* has been associated with rheumatoid arthritis (RA) and may play a role in modulating immune responses in RA patients	[50]
*Escherichia coli* (gut)	Some strains are beneficial, producing vitamin K and preventing colonization by pathogenic bacteria	Non-pathogenic *E. coli* Nissle 1917 is used as a probiotic to treat ulcerative colitis and maintain gut health	[51]
*Veillonella* (oral/gut)	Involved in lactate metabolism and may play a role in athletic performance	*Veillonella atypica* has been linked to improved endurance performance in athletes due to its role in lactate utilization	[52]

## Gene Editing Tools

Molecular techniques have revolutionized our understanding of the human microbiome, particularly with regard to modifying the genomes of gut-associated microbes. These techniques provide insights into microbial species diversity, their roles in host-microbe interactions, and how specific microbes influence health. Tools such as CRISPR-Cas9, homologous recombination (HR), and the phage-derived tools for recombinase-mediated knockouts (PTRK) system have become critical in studying and modifying microbial genomes. In this section, we explore the mechanisms of these tools, their applications in microbial genome editing, their roles in host-microbiome interactions, as well as their advantages, limitations, and current use [53, 54].

### New Advancements in Altering Microbial Genomes

**Fig. 1:**
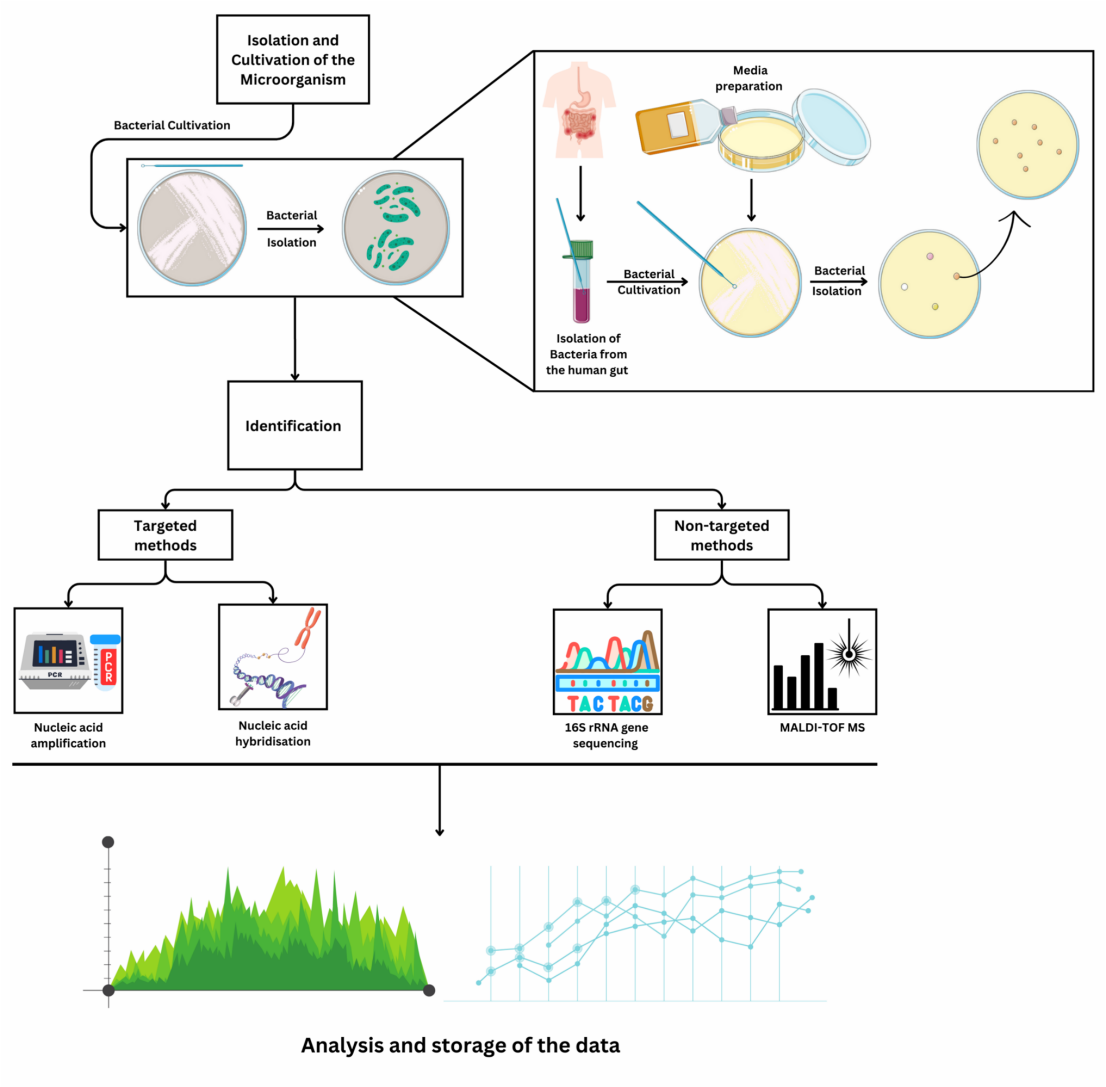
The steps in isolating, cultivating, and identifying microorganisms for microbiome-based therapies. The process begins with isolating bacteria from various environments, followed by cultivation under controlled conditions. Identification uses targeted and non-targeted methods, such as nucleic acid amplification, 16S rRNA gene hybridization, and MALDI-TOF MS analysis. These techniques allow precise microbial characterization, forming the basis for gene editing and antimicrobial peptide therapies. The generated data is systematically stored and interpreted to guide further research. Understanding microbial evolution and behavior enables unlocking the microbiome’s potential to address health challenges with next-generation treatments in health and biotechnology

Advancements in gene editing techniques have significantly improved our ability to study and manipulate microbial communities with precision. The 16S rRNA gene is one of the most commonly used phylogenetic marker genes in microbiome studies. This gene is found in nearly all bacteria and archaea, allowing researchers to classify microbial communities based on sequence similarity (Fig. 1). By sequencing 16S rRNA genes, researchers can identify microorganisms present in a sample, assess microbial biodiversity, and analyze phylogenetic relationships [55, 56].

After identifying microbiota species using high-throughput sequencing and culture testing, gene editing tools can be used to probe microbial functions and their roles in host health. Below, we outline some of the most important tools used in modifying microbial genomes.

#### HR

HR has long been a staple in genome editing. In HR, target genes are modified through sequence-specific nucleases such as RecA and RecBCD. These nucleases induce DNA repair mechanisms that allow targeted mutations to be introduced into the genome [57].

**Fig. 2:**
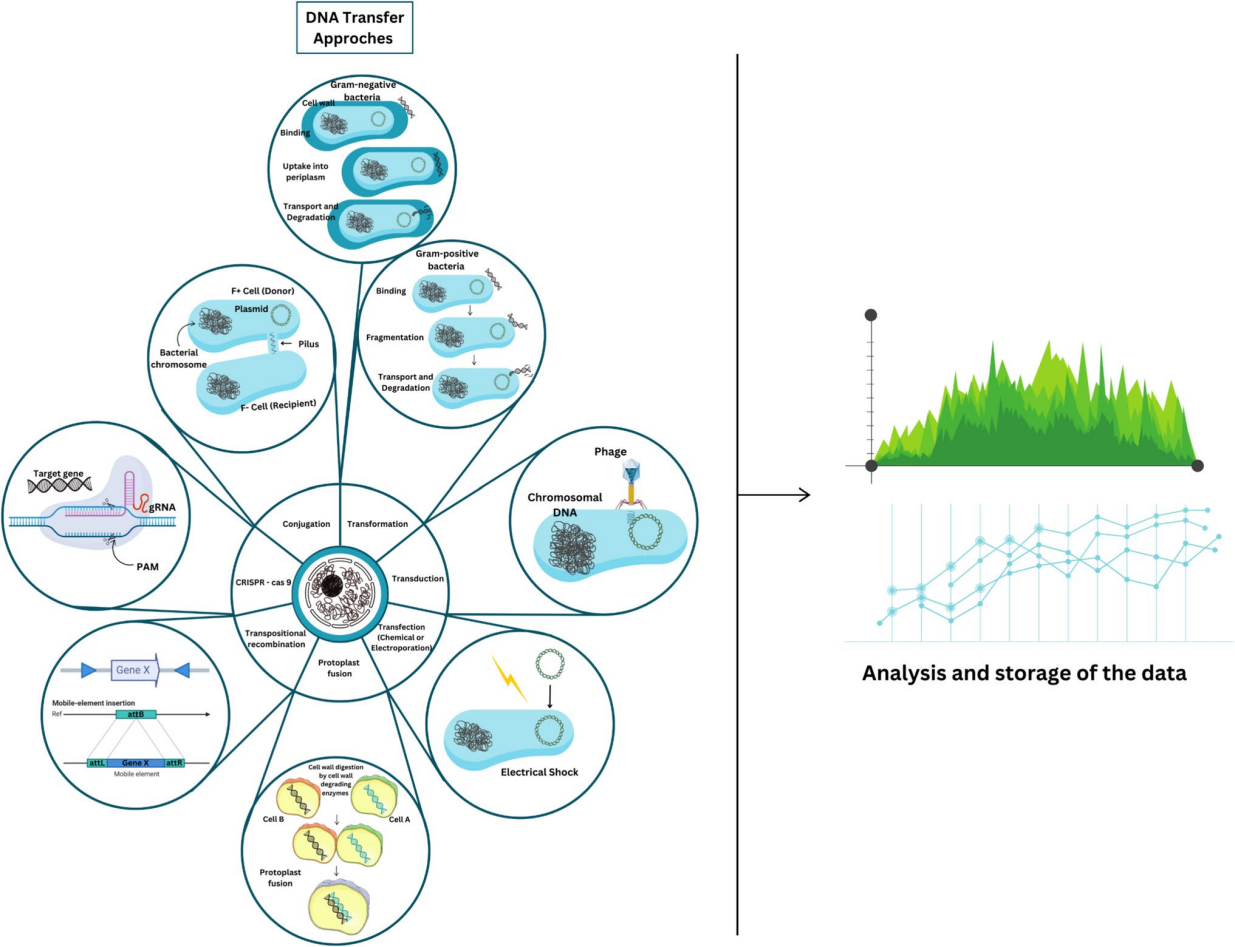
DNA transfer methods fundamental to genetic engineering, synthetic biology, and molecular research. These techniques introduce genetic material into cells, enabling genome modifications and functional studies. Conjugation transfers DNA directly between bacterial cells via a pilus, spreading genetic traits. Transformation allows cells, both Gram-positive and Gram-negative, to uptake extracellular DNA, with distinct transport and integration mechanisms for each. Transduction involves bacteriophages transferring chromosomal DNA between bacterial cells. Transfection introduces DNA through chemical methods or electroporation, altering membrane permeability. Protoplast fusion merges enzymatically treated cells for genetic recombination, commonly used in plant and microbial genetics. Transpositional recombination integrates mobile genetic elements into the genome, driving genetic variation. CRISPR-Cas9 enables precise genome editing via RNA-guided DNA cleavage. After DNA transfer, computational analysis and data storage help interpret genetic modifications. Bioinformatics tools analyze gene expression, validate edits, and assess functional impacts, advancing synthetic biology, biotechnology, and therapeutic applications

A key component of HR is the use of a counter-selectable marker and a plasmid with a selectable marker, which stabilize insertion mutations (Fig. 2). A limitation of HR is that multiple mutations cannot be introduced using the same selection marker, and insertional deactivation of target genes in operons may cause polar effects on downstream genes. A solution to this is double-crossover HR, which allows for marker-less gene deletions through plasmid integration and subsequent removal [58].

#### CRISPR-Cas9 and CoCas9

The CRISPR-Cas9 system has been a transformative tool in microbiome research. It uses a guide RNA to direct the Cas9 protein to a specific location in the DNA, where it creates a double-strand break (DSB). The cell then repairs the break through two main pathways: non-homologous end joining (NHEJ), which results in gene knockouts, or homology-directed repair (HDR), which allows for precise edits [58, 59]. Recent developments have expanded CRISPR’s applications to rapid diagnostics, enabling the identification of microbial pathogens, antibiotic-resistant strains, and the forecasting of various biological targets.

CRISPR-based bacterial detection tools are now integrated into microbiome studies to enhance pathogen surveillance and therapeutic interventions [14]. Several CRISPR-associated nucleases, including Cas9, Cas12, Cas13, and Cas14, are crucial to this diagnostic revolution, with each offering unique functionalities that contribute to their diagnostic versatility. Cas9, the most widely known CRISPR nuclease, is primarily used for DNA targeting and is incorporated into diagnostic methods like lateral flow assays and PCR-based detection due to its high sensitivity and specificity. Cas12 and Cas13 have expanded CRISPR diagnostics to RNA-based targets. Cas13, in particular, enables precise RNA interference and cleavage, enhancing signal amplification for the sensitive detection of RNA viruses such as Zika and COVID-19. Similarly, the small yet powerful Cas14 enzyme has demonstrated potential in detecting single-stranded DNA (ssDNA), providing a means to identify genetic alterations and ssDNA viruses. The CRISPR-Cas systems’ flexibility, including their integration with isothermal amplification techniques, biosensors, and fluorescence-based systems, has significantly broadened their applicability across various diagnostic fields [60].

**Fig. 3:**
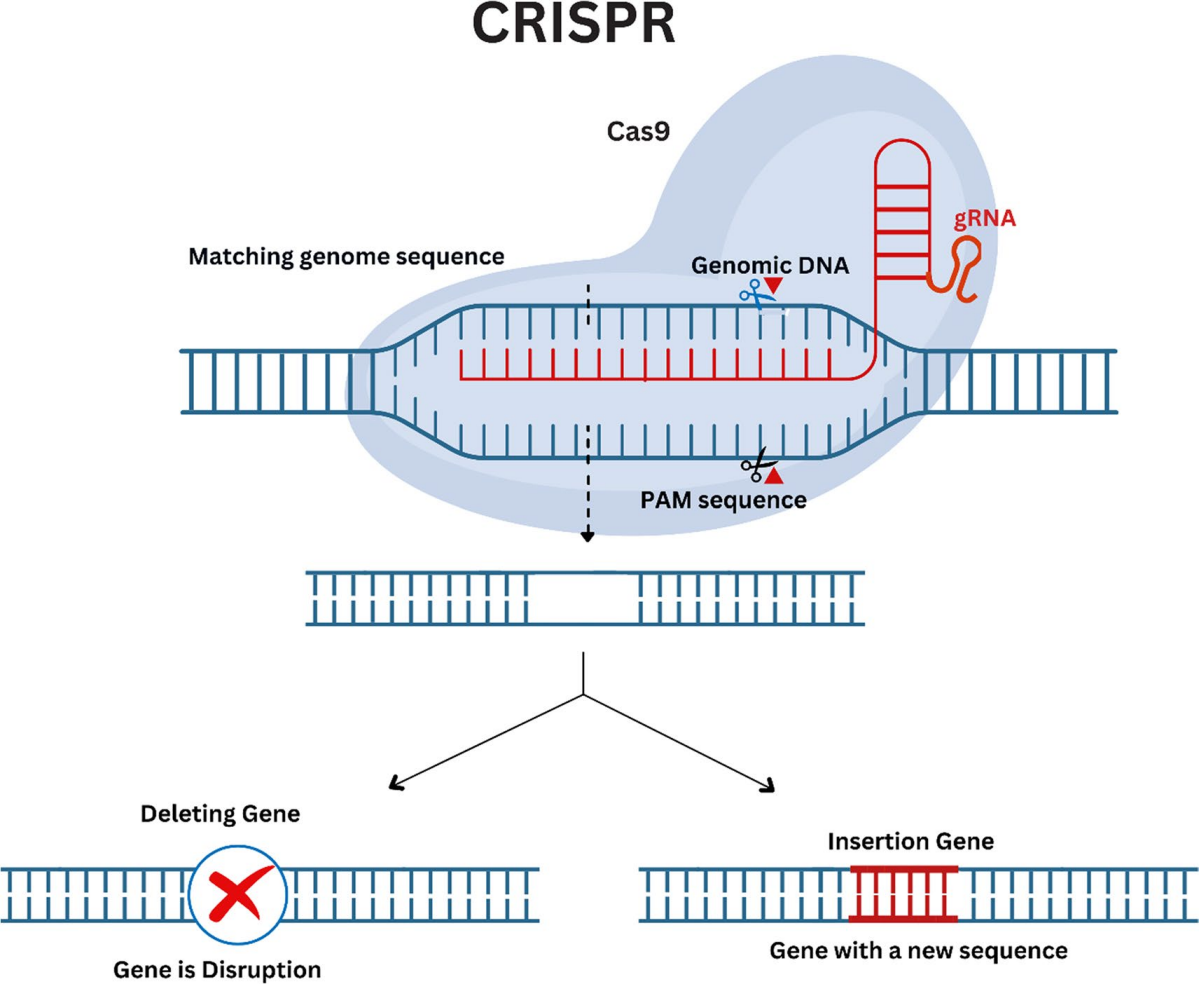
The CRISPR-Cas9 genome editing system, a tool for precise genetic modifications. The Cas9 protein, guided by RNA (gRNA), targets a specific DNA sequence. The adjacent protospacer adjacent motif (PAM) is essential for Cas9 to recognize and cleave the DNA. Once the Cas9-gRNA complex binds, the Cas9 protein introduces a double-strand break, enabling genome editing outcomes such as gene deletion, insertion of new material, gene disruption, or sequence replacement. These modifications are facilitated by the cell’s DNA repair mechanisms, allowing precise changes to the genome. The figure demonstrates the precision and versatility of CRISPR-Cas9, which has revolutionized genetic research, biotechnology, and therapeutic applications

The CoCas9 variant, derived from the human microbiome, offers unique advantages in microbial genome editing. CoCas9 is smaller than traditional Cas9 (just 1004 amino acids) and is highly effective in combating specific genome sites. CoCas9’s protospacer adjacent motif (PAM) sequences (e.g., 5'-N4GWNT-3') are particularly useful in guiding the Cas9 protein to the correct sequence for editing [59]. The ability to introduce DSBs with high specificity makes CoCas9 a powerful tool for genome editing applications (Fig. 3).

#### PTRK System

The PTRK system is an emerging tool for gene editing that uses recombineering techniques to modify bacterial genomes. By utilizing phage-derived recombinases and CRISPR-based mechanisms, the PTRK system can create targeted DNA breaks, which are repaired through recombination. This system enables precise genetic modifications such as knockouts or insertions.

Although still underexplored in microbiome studies, PTRK shows promise for editing thermophilic lactobacilli and other species. However, it faces challenges such as the difficulty of maintaining antibiotic selection markers after single-crossover recombination and the possibility of polar effects on downstream genes [57, 62].

#### CRISPRi

CRISPR interference (CRISPRi) uses a catalytically inactive version of Cas9 (dCas9) to silence genes without permanently altering the genome. This technique is valuable for studying gene function because it allows for reversible downregulation of target genes, providing insights into the effects of gene silencing without the risk of knockout-induced lethality. Unlike traditional knockout techniques, CRISPRi offers a less permanent approach, allowing gene silencing to be reversed, which is particularly useful for studying genes essential for cell survival.

Additionally, CRISPRi has a lower risk of unintended mutations compared to conventional gene knockout methods, as it does not cause double-strand breaks in the DNA. This makes CRISPRi a safer option for transient gene silencing with fewer off-target mutations. The CRISPRi system is also flexible, enabling the simultaneous silencing of multiple genes.

However, there are some limitations. High levels of dCas9 expression can be toxic to cells, and off-target effects may lead to the unintended silencing of non-target genes (Table 5). Furthermore, the CRISPRi system remains active even in the absence of induction, which could cause slower growth rates in bacteria [61, 63–65].

**Table 5:** Gene editing tools for microbial genome modification

Gene editing tool	Mechanism	Advantages	Limitations	Applications	References
Homologous recombination (HR)	- Uses sequence-specific nucleases (e.g., RecBCD, RecA) to initiate double-strand breaks (DSBs) at targeted loci	- Precise gene editing	- Polar effects on downstream genes in operons	- Gene knockouts/insertions	[57, 58]
	- Requires a plasmid with selectable/counter-selectable markers and homologous DNA regions for integration	- Marker-less gene deletions possible through double-crossover HR	-Requires antibiotic selection for stability	- Stabilization of insertional mutations	[57, 58]
CoCas9	- A compact Cas9 ortholog (1004 aa) with high fidelity and a complicated PAM sequence (5′-N4GWNT-3′, 5′-N4GCDT-3′, 5′-N4 ATDT-3′)	- Efficient and precise genome editing	- Limited by PAM sequence specificity	- Precise genome editing in human microbiome	[54, 59]
	- Forms a Cas9-sgRNA complex to target and cleave DNA at specific sites, followed by repair through NHEJ or HDR	- Compatible with AAV vectors for delivery	- Off-target effects possible	- High-fidelity DNA cleavage and repair	[54, 59]
PTRK system	- Combines phage-derived proteins, recombineering, and CRISPR to create specific DNA breaks repaired through recombination	- Precise knockouts or insertions in microbial genomes	- Limited application in human microbiome studies	- Genetic modifications in thermophilic bacteria	[57, 62]
	- Independent of transformation efficiency and suitable for thermophilic bacteria	- Marker-less gene replacement possible	- Polar effects in operons and antibiotic selection required	- Marker-less gene replacement	[57, 62]
CRISPR interference (CRISPRi)	- Uses a catalytically dCas9 to bind DNA and prevent transcription without cleaving	- Reversible gene repression	- Off-target binding and toxicity at high dCas9 levels	- Modulation of gene expression in microbiome-linked bacteria	[61, 64]
	- Targets promoter regions to modulate gene expression	- Multiplexing capability for multiple gene repression	- Silencing of non-targeted genes	- Repression of single or multiple genes simultaneously	[61, 64]
	- Widely used in microbiome-linked bacteria (e.g., *Lactococcus lactis*, *Bacteroides thetaiotaomicron*)	- Useful for essential gene studies	- Active even without induction, potentially affecting growth	- Essential gene studies and metabolic pathway analysis	[61, 64]
16S rRNA sequencing	- High-throughput sequencing of 16S rRNA genes to identify microbial taxa, quantify biodiversity, and assess phylogenetic relatedness	- Identifies microbial community composition and diversity	- Limited resolution at species/strain level	- Phylogenetic classification of gut microbiota	[53, 56]
High-throughput culturing	- Combines 16S rRNA sequencing with high-throughput culturing to identify unknown species and expand culturable species from gut microbiota	- Expands culturable species from metagenomic databases	- Labor-intensive and time-consuming	- Isolation and identification of unknown microbial species	[53, 56]

### Microbiome Sequencing Technologies

As we discussed briefly, sequencing technologies like 16S rRNA sequencing, shotgun metagenomics, and metatranscriptomics are used before altering or changing anything inside the microbiome. These technologies enable us to characterize, describe, and classify the microbiota we intend to change [66, 67].

### 16S rRNA Approach

Classifying the microbiome in terms of taxonomy and phylogeny has been extensively studied through the sequencing of the 16S ribosomal RNA subunit gene. This gene contains regions that are conserved across bacterial species, as well as hypervariable regions unique to specific genera. These regions are targeted for sequencing and used for taxonomic classification. The 16S rRNA gene sequence shows a high degree of conservation among species. The degree of conservation varies widely between hypervariable regions, with more conserved regions correlating to higher-level taxonomy and less conserved regions to lower levels, such as genus and species.

The bacterial 16S gene contains nine hypervariable regions (V1–V9), ranging from about 30 to 100 base pairs long. The 16S rRNA gene (1500 bp) is large enough for informatics purposes. The sequenced variable regions are clustered into operational taxonomic units (OTUs), providing crucial information about the taxonomic composition of microbial communities. The process involves isolating bacteria, extracting DNA, amplifying the 16S rRNA gene, sequencing a portion of the gene, and comparing the sequence to GenBank for classification [68].

### Functional Shotgun Metagenomics

Metagenomics is an advanced approach for deciphering the genetic potential of microbial communities, especially in the contexts of antimicrobial resistance (AMR) and cancer-associated microbial interactions. Unlike traditional sequencing techniques that provide taxonomic insights, functional metagenomics identifies active genes and metabolic pathways, offering a deeper understanding of microbial functionality, resistance mechanisms, and host interactions.

This approach involves untargeted sequencing of all microbial genomes in a sample. It provides insights into microbial communities'taxonomic composition, functional potential, and even whole genome sequences. In contrast, methods like 16S rRNA gene sequencing, which focus on specific marker genes, are sometimes inaccurately labeled as metagenomics since they do not capture the full genomic content of a sample.

The metagenomic workflow includes sample collection, processing, sequencing, preprocessing of sequencing reads, sequence analysis to profile taxonomic, functional, and genomic features, statistical and biological post-processing analysis, and finally validation of results [69]. Experimental biases and the intricacies of computational analysis make this approach challenging, requiring researchers to make careful decisions at every stage to ensure reliable and meaningful results [70].

#### Functional Metagenomics in Antimicrobial Resistance (AMR)

Functional metagenomics facilitates the discovery of novel antimicrobial resistance genes by cloning and expressing metagenomic DNA in heterologous hosts. This technique can identify resistance determinants that conventional culture-based or sequence-driven analyses might overlook. Recent studies have indicated that resistance genes against a broad spectrum of antibiotics, including beta-lactams, tetracyclines, and aminoglycosides, have been found in diverse environments such as soil, gut microbiota, and wastewater [71, 72]. It has also revealed previously unidentified resistance mechanisms, such as novel beta-lactamases and efflux pumps, and has clarified the role of mobile genetic elements in horizontal gene transfer [73, 74].

#### Functional Metagenomics in Cancer-Associated Microbial Interactions

The microbiome plays a significant role in cancer development, and functional metagenomics is a powerful tool for investigating the molecular mechanisms behind these interactions. Through the analysis of microbial genes and pathways, researchers have identified microbiota-derived metabolites, such as SCFAs and bile acids, that influence tumor growth, immune response, and therapy efficacy [75, 76].

Integrating functional metagenomics with multi-omics approaches, such as metatranscriptomics and metabolomics, is crucial for clarifying microbial roles in AMPs and cancer. Advances in high-throughput functional screening, synthetic biology, and AI-driven bioinformatics will further enhance the efficiency of functional gene discovery, paving the way for novel antimicrobial therapies, cancer biomarkers, and precision medicine strategies.

### Meta Transcriptomics

Complete microbial communities in a sample are analyzed using the cutting-edge -omics technique known as meta transcriptomics (Table 6). Unlike metagenomics, which examines DNA to determine the genetic potential of microorganisms, meta transcriptomics provides insight into which genes are actively expressed, revealing the real-time functioning of microbial communities [66].

**Table 6:** Gene sequencing technologies

Sequencing method	Description	Key applications	Advantages	Limitations	References
16S rRNA sequencing	Targets the 16S ribosomal RNA gene, which contains conserved and hypervariable regions for taxonomic classification	- Microbial community composition analysis—Taxonomic classification at genus/species level—Studying microbial diversity in different environments	- Cost-effective—Well-established method—High-throughput sequencing	- Limited to bacteria and archaea—Does not provide functional insights—Primer bias may affect results	[68, 78]
Shotgun metagenomics	Sequences all microbial DNA in a sample, enabling taxonomic and functional profiling	- Identifying microbial genes and metabolic pathways—Studying antimicrobial resistance (AMR)—Whole-genome reconstruction of microbes	- Provides both taxonomic and functional insights—Can detect rare and unculturable microbes—No need for PCR amplification	- High computational demand—Requires deeper sequencing coverage—Complex bioinformatics analysis	[69, 73–75]
Metatranscriptomics	Examines RNA to identify actively expressed genes, revealing real-time microbial functions	- Studying gene expression in microbial communities—Understanding functional responses to environmental changes—Identifying active metabolic pathways	- Provides insights into real-time microbial activity—Can distinguish between live and dormant microbes	- RNA is less stable than DNA - Requires rRNA depletion - High sequencing cost and complex analysis	[69, 77]

The process begins with RNA extraction, where total RNA is isolated from the sample. Next, rRNA depletion removes ribosomal RNA (rRNA) to enrich for mRNA, which carries gene expression information. Following this, cDNA synthesis converts RNA into complementary DNA (cDNA) for sequencing. The cDNA is then sequenced using high-throughput platforms (e.g., Illumina). Finally, data analysis involves mapping the sequences to reference databases to identify expressed genes, pathways, and functional activities [76].

## Specific Applications in Combating Microbes and Enhancing Beneficial Bacteria

### AMPs from the Microbiome

AMPs produced by the microbiome have become a focal point of research due to their potent antimicrobial and anti-inflammatory properties. One such peptide, lactomodulin, isolated from *Lactobacillus rhamnosus*, exhibits a broad spectrum of activity against various pathogens with minimal cytotoxicity. In laboratory tests, lactomodulin has demonstrated significant inhibitory activity against *Clostridium difficile* with a minimum inhibitory concentration (MIC50) of 0.4 µmol, and complete inhibition at a concentration of 0.9 µmol [77]. Furthermore, its activity extends to pathogens like *Salmonella enterica*, *Escherichia coli*, *Pseudomonas aeruginosa*, vancomycin-resistant *Enterococcus faecium*, *Listeria monocytogenes*, and *methicillin-resistant Staphylococcus aureus* (MRSA). Notably, lactomodulin shows its most potent effects against antibiotic-resistant MRSA, with a MIC50 of 0.2 µmol. Despite these promising results, lactomodulin exhibits limited efficacy against Gram-negative bacteria such as *E. coli*, *S. enterica*, and *P. aeruginosa*. This selectivity highlights a potential challenge in applying lactomodulin in broader clinical scenarios, especially for infections caused by Gram-negative pathogens. Additionally, lactomodulin does not show antifungal activity, even at doses 20 times higher than its typical MIC50, indicating that its therapeutic potential may be restricted to certain bacterial infections [79].

Another AMP, lugdunin, derived from *Staphylococcus epidermidis*, exhibits potent bactericidal activity against Gram-positive bacteria, including *Staphylococcus aureus*. Lugdunin works by disrupting bacterial cell membranes and acidifying the cytoplasm, which depletes the energy of *S. aureus*. This peptide also binds to small monovalent ions such as Na^+^, K^+^, and Li^+^, causing an ion imbalance that disrupts energy production in bacteria [80]. Interestingly, lugdunin’s effectiveness is influenced by membrane cholesterol concentration, with reduced activity in cholesterol-rich membranes. However, the peptide’s activity can be enhanced when membrane cholesterol levels are lowered by treatments such as trypsin [80]. These characteristics position lugdunin as a promising therapeutic candidate, particularly for combating Gram-positive bacterial infections.

Salivaricin 9, an antibiotic produced by *Streptococcus salivarius* NU10, is another AMP with potent antimicrobial properties. It targets *Corynebacterium* spp. and *Micrococcus luteus* by forming pores in bacterial membranes, as confirmed by fluorescence assays and MALDI-TOF analysis, which identified its molecular weight as 2560 Da [81]. Its bactericidal action, by compromising the integrity of the bacterial cell membrane, offers a potential strategy for controlling infections caused by these specific pathogens.

Microcin PDI (MccPDI), a peptide effective against *Shigella* and multidrug-resistant *E. coli*, provides further evidence of the diverse modes of action exhibited by AMPs. However, resistance to MccPDI has been observed in some bacterial strains due to mutations in the OmpF protein, which prevents the peptide from entering bacterial cells. Moreover, certain strains, such as *MAD 96*, produce their own antimicrobial peptides like colicins to counteract MccPDI [82]. This highlights the need for more research into the mechanisms of resistance and strategies to circumvent them. Co-culture experiments have confirmed MccPDI’s bactericidal effects, significantly reducing bacterial populations and providing insights into its therapeutic potential.

Nisin, produced by *Lactococcus lactis*, is one of the most extensively studied lantibiotics. It exhibits potent activity against Gram-positive bacteria, including *Listeria monocytogenes* and *Clostridium botulinum*, with limited resistance development. Nisin targets lipid II, a critical precursor in bacterial cell wall biosynthesis, disrupting cell wall synthesis and inducing membrane damage. However, its activity against Gram-negative bacteria is limited because lipid II is located in the inner membrane, which is inaccessible in these pathogens [83]. Researchers have been working to enhance nisin’s efficacy against Gram-negative bacteria by combining it with other antimicrobial peptides. These hybrid approaches have shown up to 12-fold enhanced activity against Gram-negative pathogens, offering a promising direction for expanding nisin’s antimicrobial spectrum [84]. Given its long history of use as a food preservative, nisin’s safety profile is well-established, making it a suitable candidate for further clinical exploration [84, 85].

### AMPs as Regulators of Microbial Defense and Homeostasis

The human body hosts a diverse microbiota that plays an indispensable role in health, influencing processes such as nutrient digestion, immune regulation, and protection against pathogens. These microbes, including bacteria, fungi, viruses, and archaea, help to maintain a delicate balance between defending against harmful pathogens and supporting the body’s immune functions. AMPs, such as bacteriocins, defensins, and cathelicidins, are critical to this balance. They act as a first line of defense, preventing infections by directly combating harmful microorganisms and modulating the immune system [86].

Moreover, AMPs produced by the gut microbiota are integral to maintaining the integrity of the gastrointestinal tract. For example, *Bacillus* and *Lactobacillus* species, which are common producers of AMPs in the human gut, produce bacteriocins and lipopeptide antibiotics that suppress the growth of pathogenic bacteria [86, 87]. These peptides not only kill harmful bacteria but also regulate the population dynamics within the microbiome, thereby preventing dysbiosis, which is linked to a range of conditions including gastrointestinal disorders, obesity, and metabolic diseases. Probiotic strains like *Lactobacillus* and *Enterococcus* are known to enhance antimicrobial activity in the intestinal lumen, helping to maintain microbial homeostasis and prevent pathogenic overgrowth [88].

Through the gut-brain axis, the microbiome also influences mental health and behavior, with increasing evidence linking it to conditions like anxiety, depression, and neurodevelopmental disorders. This underscores the profound role of AMPs not just in preventing infections, but in supporting broader aspects of human health [89].

AMPs are also key modulators of the immune system, activating immune cells such as neutrophils and macrophages and triggering immune pathways, including toll-like receptor. This activation leads to cytokine production and the modulation of inflammation, both of which are crucial for mounting an effective immune response. Additionally, AMPs selectively target harmful bacteria while allowing beneficial microbes to thrive, ensuring that microbial populations are in balance. For instance, defensins in the gut help regulate bacterial populations by selectively combating pathogenic species while promoting the growth of beneficial microbes [90].

By maintaining this microbial balance, AMPs foster mutualistic relationships between the host and its microbiota, which are vital for metabolic and immune functions. Disruption of these interactions, through a loss of AMP production or an overgrowth of pathogenic species, can lead to a range of diseases, emphasizing the importance of understanding and manipulating these peptides for therapeutic purposes. Recent advancements have focused on enhancing the production of AMPs or supplementing them with external sources, offering potential strategies for treating infections and preventing microbial imbalances associated with chronic conditions [90].

In conclusion, AMPs play a crucial role in maintaining microbial defense and homeostasis within the body. Their antimicrobial properties are complemented by their broader physiological roles, including immune modulation and maintenance of microbial balance. Ongoing research into the therapeutic use of AMPs, including their delivery mechanisms and potential for combination therapies, promises to enhance our ability to treat infections while preserving the delicate equilibrium of the human microbiome [91].

## Microbiome and Cancer: Interactions and Therapeutic Potential

### The Influence of the Microbiome on Cancer Treatment

The gut microbiota plays a pivotal role in various physiological processes, including metabolism, nutrient regulation, homeostasis, and development. It significantly influences human health, adaptive immune responses, and the effectiveness of cancer treatments, including immune checkpoint inhibitors [92, 93]. Notably, the microbiota’s influence extends to inflammation and the body’s response to tumor therapies, interacting with both immune and metabolic pathways [94]. With cancer being a leading cause of death globally [95], recent studies have shed light on the vital role of specific bacterial species in inhibiting tumor growth. Bacteria such as *Faecalibacterium* and *Clostridiales* from the *Firmicutes* phylum have been found to exert anti-inflammatory and anti-tumorigenic effects, contributing to tumor suppression and providing new therapeutic avenues [96].

Clinical investigations further emphasize the potential of gut microbiota in enhancing cancer treatment efficacy. One key mechanism by which the microbiome influences therapeutic outcomes is by modulating immune responses. For instance, certain beneficial bacteria, including *Bifidobacterium* and *Akkermansia muciniphila*, can boost the activity of dendritic cells and promote T-cell infiltration into tumors, improving the effectiveness of programmed cell death protein-1 (PD-1) blockade therapy in advanced solid tumors [97]. This highlights how microbiota modulation could be leveraged to enhance the response to immunotherapies.

Furthermore, the gut microbiota acts as an immune booster by stimulating T-cell responses, which can help fight cancer. These findings suggest that the composition of the microbiome may not only serve as a predictive biomarker but could also become a therapeutic target for cancer treatments. Metabolic by-products of gut bacteria, such as butyrate, SCFAs, and propionate, play a crucial role in cancer therapy. These metabolites promote anti-tumor immunity, support T-cell differentiation, reduce tumor-associated inflammation, and create an environment that enhances the efficacy of cancer treatments [98].

The microbiota also plays a vital role in mitigating chemotherapy-induced toxicity. It helps restore epithelial barrier function and modulates systemic inflammation, thus alleviating side effects and improving the patient’s quality of life during treatment [99]. Modulating the gut microbiota through approaches such as prebiotics, probiotics, and FMT is increasingly seen as a promising strategy in cancer therapy (Table 7). For example, probiotic strains like *Lactobacillus* have been shown to boost the effectiveness of immune checkpoint blockade (ICB) therapy by stimulating cytokine secretion and activating natural killer (NK) cells, which contribute to anti-tumor immunity [100].

**Table 7:** Microbiome species and their roles in cancer therapy

Microbiome species	Role in cancer therapy	References
*Faecalibacterium*	Anti-inflammatory, anti-tumorigenic effects	[96]
*Bifidobacterium*	Enhances immune checkpoint blockade (ICB) efficacy, increases T-cell infiltration	[97]
*Akkermansia muciniphila*	Improves response to PD-1 blockades in advanced solid tumors	[97]
*Clostridiales*	Inhibits tumor growth through anti-inflammatory effects	[96]
*Lactobacillus*	Enhances ICB therapy, stimulates cytokine secretion, activates NK cells	[100]

### Gene Editing and AMP-Based Microbiome Therapeutics in Cancer

#### Anticancer Properties of AMPs

AMPs have emerged as promising agents in cancer treatment, particularly due to their ability to selectively target cancer cells through interactions with cell membranes. These peptides generally possess a positive net charge, which makes them particularly attracted to the negatively charged membranes of cancer cells [101]. As a result, AMPs can be used to disrupt cancer cell membranes and target specific intracellular processes, offering a therapeutic approach that can bypass some of the limitations of conventional treatments.

Peptide-based therapies have seen significant commercial success, with over 80 peptide-based medications currently approved for treating a range of diseases, including cancer, diabetes, and osteoporosis [102]. In the realm of cancer, AMPs and anticancer peptides (ACPs) share structural features such as a positive charge, amphipathic nature, and hydrophobicity, which enhance their interaction with cell membranes. These characteristics have led to ongoing research into the anticancer potential of AMPs. Cancer cells, having more negatively charged membranes, are particularly susceptible to the action of ACPs, making them an effective therapeutic target that is less likely to develop resistance [103].

Several AMPs have shown promising therapeutic efficacy in preclinical cancer models. For instance, *Microcin E492* from *Klebsiella pneumoniae* and the scorpion-derived *BmKn2* peptide have demonstrated anticancer activity by altering cellular pathways, disrupting cell membranes, and directly inhibiting tumor growth [104]. Notably, the BR2 peptide has exhibited cytotoxic effects on human cervical and colon cancer cells in vitro, and its anticancer activity has been confirmed in vivo in a mouse melanoma model [101]. Peptides such as BR2, pAntp, and pTAT, which are classified as cell-penetrating peptides (CPPs), have also gained interest due to their ability to facilitate the delivery of therapeutic compounds into cells [105, 106]. When combined with chemotherapeutic agents like 5-fluorouracil, CPPs like KT2 [107] and RT2 [108] have shown increased effectiveness against metastatic colon cancer cells.

### Clinical Studies on Microbiome-Based Therapies

AMPs have been the focus of several clinical trials, particularly in cancer therapy and the treatment of antibiotic-resistant infections. Peptides such as LL37 and LTX-315 have shown promise in early-phase trials (Table 8) [101]. For example, a phase II clinical trial (NCT02225366) assessed the efficacy of the AMP LTX-315 in patients with melanoma. The study found that intratumoral administration of LTX-315 induced immunogenic cell death (ICD) and enhanced the antitumor immune response, resulting in significant tumor regression in a subset of patients. In another phase I/II trial (NCT01058616), the AMP LL-37 was tested in patients with chronic wounds infected with multidrug-resistant pathogens. The trial reported accelerated wound healing and reduced bacterial load, suggesting that LL-37 could be a promising alternative to traditional antibiotics in managing chronic infections. Ongoing research is exploring the potential of AMPs in combination therapies. For instance, a clinical trial (NCT04796194) evaluated the efficacy of combining AMPs with immunotherapeutic agents like pembrolizumab in patients with aggressive cancers, including triple-negative breast cancer and melanoma (Table 9). This study aims to determine if the synergistic effects of AMPs and immune checkpoint inhibitors can enhance anti-tumor immune responses and improve clinical outcomes in these challenging malignancies [109].

**Table 8:** Antimicrobial peptides (AMPs) with anticancer properties

AMP name	Source	Mechanism of action	Cancer type targeted	References
LL37	Human	Disrupts cancer cell membranes, induces immunomodulation	Melanoma, breast cancer	[101]
LTX-315	Synthetic	Lyses tumor cells, releases tumor antigens	Melanoma	[101, 110]
BR2	Synthetic	Cytotoxic to cancer cells, penetrates cell membranes	Cervical, colon cancer	[101, 110]
BmKn2	Scorpion-derived	Alters intracellular pathways, ruptures cell membranes	Colon, bladder cancer	[104]
Microcin E492	*Klebsiella pneumoniae*	Prevents tumor cell growth, disrupts cell membranes	Colon cancer	[104]

**Table 9:** Summary of recent clinical studies on microbiome-based therapies

Clinical trial ID	Therapy	Condition	Key findings	Reference
NCT02225366	LTX-315 (AMP)	Melanoma	Induced immunogenic cell death and tumor regression	[111]
NCT01058616	LL-37 (AMP)	Chronic wounds with MDR pathogens	Accelerated wound healing and reduced bacterial load	[112]
NCT04796194	CRISPR-engineered *Lactobacillus rhamnosus* GG	Triple-negative breast cancer	Enhanced tumor regression and CD8 + T cell infiltration	[109]

### Mechanism of Action of Various AMPs

AMPs are known for their ability to fight infections by disrupting microbial cells in various ways, and many of these mechanisms are directly applicable to cancer therapy. The primary mode of action involves the disruption of microbial cell membranes, which are typically negatively charged, leading to cell death. Additionally, AMPs can inhibit the synthesis of proteins, RNA, and DNA, further contributing to their antimicrobial effects. Some AMPs can even interact directly with intracellular components, further enhancing their therapeutic potential [113].

Among the most studied AMPs, human neutrophil peptide 1 and human β-defensin 3 are noteworthy for their ability to cross cell membranes without causing disruption (Table 10). These peptides also have significant immunomodulatory functions, promoting macrophages to secrete important cytokines like tumor necrosis factor-alpha and interferon-gamma, which are critical for mounting an effective immune response [114–116]. This makes them particularly attractive as immunomodulatory agents in cancer therapy, where enhancing immune responses is crucial for treatment success.

**Table 10:** Microbiome modulation strategies in cancer therapy

Strategy	Mechanism	Clinical applications	References
Probiotics	Stimulates cytokine secretion, activates NK cells, enhances ICB therapy	Improves response to immunotherapy	[100]
Prebiotics	Promotes growth of beneficial bacteria, enhances SCFA production	Reduces tumor-associated inflammation	[98]
FMT	Restores gut microbiota diversity, modulates systemic inflammation	Reduces chemotherapy-induced toxicity	[99]
Dietary interventions	Alters gut microbiota composition, enhances antitumor immunity	Improves the efficiency of cancer therapies	[97]

**Fig. 4:**
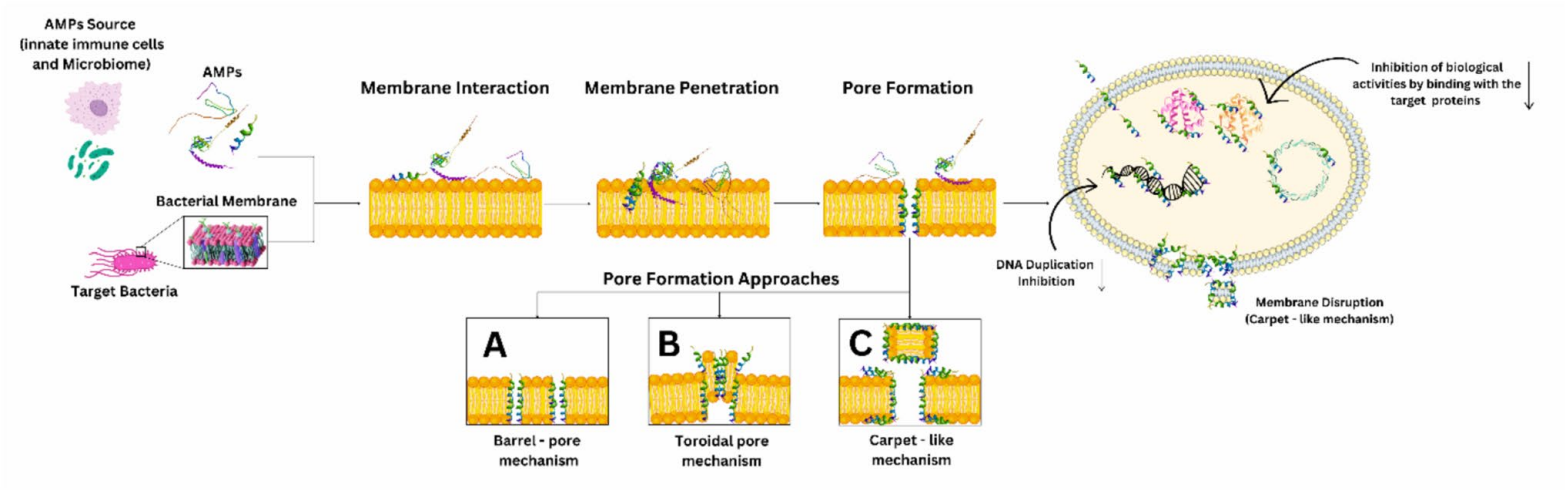
The mechanism of action of antimicrobial peptides (AMPs) derived from innate immune cells and the microbiome on bacterial membranes. AMPs interact with the bacterial membrane, leading to penetration through mechanisms such as the barrel-pore, toroidal pore, or carpet-like mechanisms. This results in pore formation that disrupts membrane integrity, inhibiting DNA replication and other biological activities by binding target proteins, ultimately causing bacterial cell death. The figure outlines the key steps: membrane interaction, penetration, pore formation, and the initiation of antimicrobial action

Other AMPs, such as buforin-II, exert their antimicrobial effects by binding directly to DNA and RNA, thereby interfering with cellular processes that lead to cell death [117, 118]. Similarly, the peptide indolicidin works by binding to double-stranded DNA, inhibiting transcription and replication, which halts microbial growth [119]. This direct interaction with genetic material suggests that such peptides could potentially be used to target cancer cells in a similar manner, offering a promising strategy for the development of novel anticancer therapies (Fig. 4).

## Development of Engineered Probiotics Producing AMPs with Anticancer Properties

AMPs have garnered significant attention in recent years for their potential role in cancer therapy. One of the most promising advancements has been the repurposing of AMPs into ACPs specifically designed to target cancer cells. This innovative approach not only simplifies the identification of ACPs by eliminating the need for exhaustive empirical screening but also facilitates the development of therapeutic agents capable of directly damaging critical cellular components such as nuclear and plasma membranes. This targeted action significantly reduces the likelihood of resistance, making ACPs a promising therapeutic class for cancer treatment. ACPs significantly reduce the likelihood of resistance compared to other therapies because they target multiple cellular components, such as the nuclear and plasma membranes, in a rapid and non-specific manner. This multitarget mechanism makes it harder for cancer cells to develop resistance, unlike conventional therapies that may focus on a single target or pathway. Additionally, ACPs often induce a robust immune response, which further contributes to their resistance to escape mechanisms.

A notable example is MAD1, which initially showed antibacterial activity against *Mycobacterium tuberculosis*. However, MAD1 also exhibits remarkable selectivity for ovarian cancer cells, potentially through its ability to interact with specific receptors or pathways overexpressed in ovarian cancer. Research suggests that MAD1’s ability to selectively target cancer cells is due to its unique peptide structure, which preferentially binds to cell surface markers found in ovarian cancer cells, enhancing its cytotoxicity while minimizing effects on healthy cells. Studies have confirmed that MAD1 can enhance the effectiveness of conventional chemotherapy, even in drug-resistant cancers, suggesting that ACPs derived from AMP templates could be a powerful tool in combination therapies [120].

Furthermore, a groundbreaking strategy in cancer treatment involves the use of gene-edited bacteria to deliver these AMPs directly to tumor sites. In addition to the inherent advantages of AMPs and ACPs, another innovative approach involves the use of gene-edited probiotics as carriers for delivering these therapeutic peptides directly to tumor sites. These engineered probiotics can be designed to produce and secrete ACPs, thus combining the benefits of antimicrobial peptides with targeted tumor therapies. This approach optimizes therapeutic effects while minimizing systemic toxicity, allowing for more targeted and efficient treatments.

### Gastric Cancer and Helicobacter pylori: A Case Study of Engineered Probiotics

Gastric cancer, often linked to *Helicobacter pylori* infection, underscores the pressing need for effective therapeutic interventions. In a recent study, engineered probiotics, specifically *Lactococcus lactis*, were modified to express guided antimicrobial peptides (gAMPs) combating *H. pylori*. The goal was to design gAMPs that would minimize toxicity to beneficial bacteria while effectively combating *H. pylori* in the stomach. Three distinct AMPs—alyteserin, laterosporulin, and cathelicidin-related antimicrobial peptide—were tested for their antimicrobial efficacy against *H. pylori* compared to Gram-positive bacteria. These gAMPs were introduced into *L. lactis* through a shuttle vector (pTKR) under an acid-inducible promoter, ensuring their optimal activity in acidic environments such as the stomach.

In vitro co-culture assays demonstrated that *L. lactis* producing gAMPs significantly inhibited *H. pylori* growth, with varying levels of toxicity depending on the AMP used. Importantly, the probiotic strain alone did not affect *H. pylori* growth, suggesting that the antimicrobial activity was primarily due to the AMPs. Further in vivo experiments in healthy mice with an intact microbiota showed that the engineered probiotics were able to reduce *H. pylori* loads and alleviate gastrointestinal symptoms, highlighting the therapeutic potential of engineered probiotics in microbial infection control and suggesting their broader applications in cancer therapy [121].

### CRISPR-Mediated Modulation of Microbiome Composition to Enhance Cancer Immunotherapy

An exciting frontier in cancer therapy involves the use of CRISPR/Cas9 technology to modify the TME and enhance the effectiveness of immunotherapy. One groundbreaking approach involves a self-driven CRISPR/Cas9 nanosystem that targets immunosuppressive regions of tumors, particularly those that are hypoxic. This system delivers CRISPR/Cas9 directly to tumor cells to knock down indoleamine 2,3-dioxygenase-1 (IDO1), a key immunosuppressive mediator often upregulated in aggressive cancers like triple-negative breast cancer. In this innovative method, *Lactobacillus rhamnosus GG* (LGG) serves as an effective carrier for the CRISPR/Cas9 system.

Upon activation by ultrasound (US) irradiation, the CRISPR/Cas9 nanosystem releases damage-associated molecular patterns (DAMPs) such as heat shock protein 70 (HSP70), calreticulin, and high-mobility group box 1, which collectively induce immunogenic cell death (ICD). In vitro experiments demonstrated that co-incubating 4 T1 breast cancer cells with this CRISPR/Cas9 system under ultrasound stimulation led to significant ICD and inhibited tumor cell proliferation. In addition to facilitating the delivery of CRISPR/Cas9, LGG plays a critical role in promoting immune cell infiltration and activation within the tumor, further amplifying the immune response. This pioneering CRISPR/Cas9-based system represents a novel strategy for reprogramming the TME and holds significant promise for the development of synergistic cancer treatments that combine gene editing with immunotherapy [122].

### Gene-Edited Bacteria to Deliver AMPs and Drugs Directly to Tumor Sites

A major challenge in cancer therapy lies in delivering therapeutic agents like AMPs directly to the tumor site in a controlled and efficient manner. Gene-edited bacteria have emerged as a novel solution to this problem, offering the potential for targeted delivery of AMPs and other drugs directly to tumor cells. By engineering bacteria to carry AMPs, researchers can ensure the stability of these peptides and enable their gradual release at the tumor site, optimizing their therapeutic impact.

A new strategy involves the use of synthetic bacterial vesicles (SBVs), which are more efficient than traditional bacterial outer membrane vesicles (OMVs) in delivering therapeutic agents. Unlike OMVs, which are produced in low yields, SBVs can be synthesized up to 40 times more efficiently using a specialized method. These SBVs are versatile, capable of encapsulating therapeutic agents like indocyanine green (ICG) to induce immune-stimulating cell death and catalase to alleviate tumor hypoxia. The encapsulation of these agents activates the cyclic GMP-AMP synthase/stimulator of interferon genes (cGAS/STING) pathway, enhancing the immune response against cancer cells. Moreover, a biocompatible polydopamine shell around the SBVs reduces the toxicity of lipopolysaccharides while preserving their immunostimulatory properties, making them safer for use in therapeutic applications.

When used in conjunction with photothermal and photodynamic therapies, SBVs encapsulated with ICG have been shown to successfully reprogram the TME, enhancing dendritic cell maturation and promoting CD8 + T cell responses. This innovative delivery platform holds great promise for overcoming the limitations of current cancer treatments, especially in the treatment of"cold"tumors, which typically exhibit poor immune responses. By enabling the targeted delivery of AMPs and other therapeutic agents directly to tumor sites, this approach has the potential to revolutionize cancer treatment, offering a more precise and effective alternative to conventional therapies [123].

## Ethical, Regulatory, and Ecological Considerations in Microbiome Manipulation and AMP Development

As research into microbiome manipulation advances, particularly through gene editing and the development of AMPs, ethical and ecological considerations become increasingly important. While AMPs offer exciting potential in therapeutic applications, such as managing infections like MRSA and supporting wound healing, they also introduce challenges that need to be addressed to ensure their safe and responsible use.

### Bioethical Concerns in Engineered Microbiomes

Rapid advancements in genetic engineering technologies, driven by innovations like CRISPR-Cas, have enabled extensive applications, such as modifying bacteria and other microbes to enhance human health, agriculture, and environmental sustainability. However, these developments also present significant risks, particularly to human life. The rapid pace of these advancements raises crucial bioethical concerns that require careful consideration. In 2018, Chinese biophysicist Jiankui He used CRISPR to modify the CCR5 gene in embryos, aiming to confer resistance to HIV. This controversial and unethical action sparked widespread discussion within the scientific community about the long-term implications of genetic modification on human life. As a result, stricter regulations have been introduced regarding genetic engineering in humans [124].

One significant risk is horizontal gene transfer (HGT), where genetically modified microbiomes may transmit engineered genes to neighboring microbiomes. This transfer could potentially lead to various disorders, such as autoimmune diseases, neurodegenerative conditions, metabolic diseases, allergies, cancer, infertility, and neurodevelopmental or behavioral disorders. The unintended genetic alterations that may occur raise concerns over unknown side effects, including altered microbial interactions, antibiotic resistance, and unforeseen human risks. Moreover, the long-term ecological impact of introducing synthetic microbiomes into natural ecosystems remains largely uncertain. These modified organisms could disrupt ecological balance, outcompete native species, or introduce harmful traits that negatively affect the environment.

### Horizontal Gene Transfer and Their Role in Disease Development

Genetically engineered microbiomes are designed to produce specific modified products; however, their long-term implications for human health and metabolism remain unclear. Over time, they may affect metabolic pathways by altering microbial gene expression, potentially leading to the overexpression or downregulation of certain genes. This imbalance could result in the generation of unwanted or hazardous metabolites, contributing to metabolic diseases or other health complications [125].

The hazardous substances produced by genetically modified microbiomes can decrease their efficiency by increasing clearance through immune responses. Due to these negative effects, the practical application of genetically engineered microbiomes is limited. Additionally, strict legislation and regulations govern the use of naturally existing bacteria and prohibit the application of genetically modified microbiomes.

Several obstacles limit their utilization: (1) limited release of genetically inserted genes from the microbiome; (2) uncertainty regarding the stability of the colonized modified microbiome and the continuous synthesis of necessary products in the targeted tissue; (3) ensuring effective and beneficial interactions with the enteric intestinal microbiome to enhance the microbiome-dysbiome ratio; and (4) the need for elimination after achieving the desired effect. Therefore, maintaining stability under standard laboratory and physiological conditions—both in vitro and in vivo is crucial [126].

### Unintentional Risks and Regulatory Challenges of Microbial-Engineered Products in Biotechnology

Unintentional gene alterations, including the introduction of antibiotic resistance genes (ARGs) and MDR genes, pose significant concerns. The increasing prevalence of ARGs in microbial populations presents a major public health challenge, as antibiotic resistance is widely recognized as a critical threat to global healthcare systems. Other emerging hazards include bacterial resistance to phages, medication resistance in cancer treatments, and the transfer of resistant genes through the food chain. Research has identified ARGs in commonly consumed probiotic bacteria, such as *Lactobacillus reuteri*, raising concerns about the unintentional transmission of resistance via dietary sources. Recent studies also show elevated levels of ARG carriage in *Enterobacterales* and diarrheagenic *Escherichia coli* in donors being evaluated for fecal FMT [127].

The use of microbial-engineered enzymes in food production has increased significantly, offering benefits such as improved yield, enhanced quality, and greater sustainability. However, concerns about their safety have been raised in numerous studies. Research increasingly suggests that some food additives, such as microbial enzymes, should be reassessed under the Generally Recognized as Safe GRAS classification. A key concern is the potential post-translational modifications caused by genetically modified microbiome-derived enzymes, which could alter protein structures and provoke immune responses or allergic reactions in susceptible individuals.

Microbial transglutaminase (mTG), a well-studied enzyme commonly used as a food-processing ingredient, illustrates this issue. While mTG was designated as a processing aid and granted GRAS certification decades ago, its safety remains debated. Recent studies suggest that mTG and its protein complexes may have pro-inflammatory, immunogenic, allergenic, and potentially pathogenic properties. Specific research has linked mTG to the pathophysiology of celiac disease, as it resembles human tissue transglutaminase, an enzyme involved in gluten intolerance and inflammatory processes. Furthermore, microbial transglutaminase has been identified as a bacterial survival determinant, raising concerns about the potential for HGT among microbial populations. The possibility of HGT-mediated spread of the mTG gene within the gut microbiome warrants further investigation to assess its impact on human health and microbial ecology [128].

### Ecological and Environmental Impact

Manipulating microbial communities through gene editing and AMP applications could have unintended ecological consequences. Altering microbial populations, whether in human microbiomes or agricultural settings, may disrupt microbial diversity and lead to dysbiosis. These shifts could affect broader ecosystems, making it essential to implement monitoring strategies that track the long-term effects of these interventions on microbial ecosystems. Regulatory frameworks must also evaluate and mitigate potential ecological risks associated with microbiome manipulation.

### Regulatory Considerations in Microbiome Gene Editing and AMP Development

The rise of antibiotic resistance globally has led to increased interest in AMPs as promising alternatives to traditional antibiotics. AMPs operate through diverse mechanisms, such as disrupting microbial membranes and combating essential intracellular functions, making them highly effective for treating bacterial infections, including multidrug-resistant strains. However, the development of AMPs and microbiome gene editing technologies brings forward significant regulatory challenges that need to be addressed to ensure their safe use in clinical and agricultural settings. Regulatory frameworks must consider various factors to assess the safety and efficacy of AMPs, including peptide stability, potential toxicity, and the cost of production. One important concern is the synergistic effects of AMPs when used alongside traditional antibiotics, particularly in the context of antibiotic resistance. For example, using AMPs in combination with antibiotics could either enhance their effectiveness or, conversely, promote resistance if not carefully managed.

Personalized medicine approaches, which tailor treatments to individual microbial profiles, further complicate the regulatory landscape. This variability between patients means that regulatory agencies must develop systems that are dynamic enough to adapt to evolving research and technology, allowing for both innovation and public health protection. Moreover, there is a need for continuous monitoring of AMPs’ environmental impacts, especially regarding microbial diversity. The widespread use of AMPs may inadvertently disrupt ecosystems by affecting non-target microbial communities. Regulatory bodies must develop frameworks that balance the benefits of AMPs as therapeutic agents with the need to preserve the natural microbiome and its ecological role.

In addition, the safe integration of microbiome gene editing technologies requires thoughtful regulation to prevent unintended ecological consequences. The manipulation of microbial communities, whether in human microbiomes or agricultural systems, raises concerns about unforeseen consequences on ecosystems, potentially leading to the loss of beneficial microorganisms or the emergence of harmful microbial strains [129].

Addressing these concerns through adaptive, transparent regulatory systems will be crucial to the responsible development of AMPs and microbiome gene editing technologies.

### Challenges in AMP Stability, Delivery, and Resistance Mechanisms

Despite their immense potential, the development and administration of AMPs face several challenges, particularly regarding stability, delivery, and resistance mechanisms. Oral administration of AMPs is particularly challenging due to their low bioavailability. In the digestive system, AMPs are susceptible to enzymatic breakdown, and they also face difficulties in penetrating the intestinal mucosa, limiting their effectiveness for gastrointestinal infections [130–132].

Additionally, systemic administration is hindered by rapid clearance from the bloodstream through the liver and kidneys, as well as breakdown by circulating proteolytic enzymes, limiting their therapeutic utility in internal infections [130, 131]. As a result, most efforts in AMP development have focused on creating topically applied treatments for skin and wound infections. However, several strategies are being explored to improve the stability and delivery of AMPs for in vivo therapies. Cyclizing peptides through amide or disulfide bonds enhances their structural stability, making them less prone to proteolytic degradation. Heterodetic cyclization, which forms disulfide bridges between cysteine side chains, is commonly used to stabilize AMPs [133, 134].

Modifying AMPs by adding chemical groups to the N- or C-terminus, such as acetylation or amidation, can increase their resistance to proteolysis, thereby improving their stability and efficacy in vivo [135, 136]. Incorporating non-standard amino acids, including D-amino acids, can enhance the stability, selectivity, and bioavailability of AMPs. D-amino acids, in particular, are less susceptible to enzymatic degradation, as proteolytic enzymes typically recognize only the L-forms of amino acids [137, 138]. Conjugating AMPs to nanoparticles can improve their stability, controlled release, and targeted delivery. Nanoparticles such as titanium dioxide, silver oxide, and zinc oxide also possess intrinsic antibacterial properties that may complement the antimicrobial action of AMPs, reducing the likelihood of resistance [139, 140].

While these approaches have shown promise in preclinical studies, ongoing research is necessary to optimize AMP formulations for clinical use.

### Synergy Between AMPs and Antibiotics

While pathogens may eventually develop resistance to AMPs, these peptides remain a promising option when combined with conventional antibiotics. Studies have shown that AMPs can synergize with antibiotics through several mechanisms. First, AMPs enhance membrane permeability by disrupting bacterial membranes, which allows antibiotics to penetrate more effectively and reach their intracellular targets. Additionally, AMPs can destabilize biofilm structures that protect bacteria from antibiotic action, which is particularly important in chronic infections where biofilms are a common barrier to treatment. Furthermore, AMPs can boost the efficacy of antibiotics, particularly those that target intracellular processes, by facilitating better access to their action sites.

In vivo studies also highlight additional benefits of combining AMPs with antibiotics. AMPs can promote tissue healing and regeneration, improving therapeutic outcomes. They can also modulate immune responses, reducing inflammation and speeding recovery from infections. Moreover, AMPs stimulate immune system activity, supporting the body’s ability to combat infections.

AMPs are particularly effective in overcoming bacterial resistance mechanisms, such as efflux pumps that actively expel antibiotics from bacterial cells. By destabilizing the bacterial membrane, AMPs allow antibiotics to accumulate inside the cell, circumventing these protective mechanisms. Additionally, AMPs are effective in treating biofilm-associated infections by disrupting biofilm matrices, making bacteria more susceptible to both AMPs and antibiotics. This combined effect is especially useful in chronic infections, where biofilms often protect bacteria from conventional treatments [129].

## Research Gaps and Future Directions

While the research into microbiome-based therapies, especially in the context of AMPs and gene editing technologies, has made significant strides, several key research gaps remain. Addressing these gaps will be essential to unlocking the full therapeutic potential of these innovative approaches.

### Ecological Impact and Long-Term Consequences of Microbiome Manipulation

One of the most significant gaps in current microbiome research is the ecological impact of gene-edited microbes, engineered probiotics, and AMP therapies on the gut and broader microbiome. The microbiome is an incredibly dynamic and diverse ecosystem, with complex interdependencies between microbial species. Introducing gene-edited organisms or AMPs could have unintended consequences that disrupt microbial homeostasis. For instance, the overgrowth of certain engineered strains could lead to dysbiosis, which has been linked to various health conditions, including IBD and metabolic disorders.

To address these concerns, future research should focus on the long-term ecological impact of microbiome-based interventions. This includes studying the durability of gene-edited organisms in the microbiome, their interaction with resident species, and any unintended side effects. Additionally, more research is needed on the resilience of microbiomes after such interventions, as well as on the ability to restore a balanced microbiome should disruptions occur. This could involve longitudinal studies in humans and animal models to monitor the sustained impact of these interventions on microbiome composition, functionality, and health outcomes.

### Optimization of AMP Stability, Bioavailability, and Delivery Systems

While AMPs show great promise as therapeutic agents, their application faces significant barriers related to stability, bioavailability, and targeted delivery. AMPs are highly susceptible to degradation by proteolytic enzymes, poor absorption through the gut, and rapid elimination from the body, particularly through the kidneys and liver. These challenges limit their efficacy when administered orally or systemically.

There is a critical need for the development of novel formulations that enhance the stability and bioavailability of AMPs. Approaches such as nanoparticle encapsulation, liposomal formulations, and peptide conjugation to carrier molecules (e.g., polymers or antibodies) could help protect AMPs from degradation and enhance their delivery to target sites. Additionally, strategies like peptide cyclization, modifications of amino acids (e.g., D-amino acids), and the incorporation of unnatural amino acids can be explored to improve resistance to enzymatic degradation, while maintaining antimicrobial activity.

Furthermore, the creation of targeted delivery systems is essential to increase the therapeutic efficacy of AMPs. For example, coupling AMPs to smart delivery vehicles, such as polymeric nanoparticles or hydrogel-based systems, could provide controlled release of peptides at the site of infection or cancer cells, minimizing off-target effects and improving patient outcomes.

### Personalized Microbiome-Based Therapeutics

The idea of personalized medicine based on microbiome composition is an emerging frontier in microbiome-based therapies. Every individual has a unique microbiome, shaped by genetic, environmental, and lifestyle factors. These differences can have a significant impact on the effectiveness of microbiome-based therapies, such as AMPs and gene-edited probiotics. A one-size-fits-all approach to treatment may not be effective for everyone, making personalized treatments an essential goal.

To move toward personalized microbiome therapies, future research should focus on the integration of metagenomic, proteomic, and metabolomic data to better understand how individual microbiomes respond to AMPs and gene-edited probiotics. Using machine learning algorithms to analyze large-scale microbiome data could help identify biomarkers associated with positive treatment outcomes, guiding personalized interventions. Furthermore, biomarker-driven clinical trials will be necessary to identify how variations in microbiome composition influence the effectiveness of AMP-based therapies. The design of microbiome-tailored treatments will require an interdisciplinary approach, combining microbiology, bioinformatics, and clinical research. By understanding the microbiome’s unique response to specific AMPs, it will be possible to create more precise and effective treatments for various diseases, from infections to cancer.

### Antimicrobial Resistance and Synergistic Approaches

As AMR continues to rise, the role of AMPs as alternatives or adjuncts to conventional antibiotics becomes even more critical. However, the development of resistance to AMPs remains a significant concern. Pathogens may evolve mechanisms to evade AMP activity, just as they have with traditional antibiotics. Moreover, while AMPs show great promise in vitro, their efficacy in vivo often depends on the context, and challenges such as biofilm formation or altered immune responses can undermine their effectiveness.

Research should focus on understanding the mechanisms of resistance to AMPs, including efflux pumps, membrane modifications, and peptide degradation by bacterial proteases. Identifying strategies to overcome AMP resistance, such as combining AMPs with conventional antibiotics or other AMPs in a synergistic fashion, is a key research direction. Studies investigating AMP-antibiotic combinations and their synergistic effects will help in overcoming AMR and improve treatment outcomes, especially in the context of chronic infections. Additionally, exploring how AMPs can disrupt biofilms—which are common in chronic infections—could help overcome one of the most significant barriers to the effective treatment of these infections. Incorporating biofilm-disrupting agents into AMP therapies could further enhance their efficacy.

### Ethical, Legal, and Regulatory Challenges in Microbiome Gene Editing

Gene editing technologies, such as CRISPR, have revolutionized microbiome research, but their application in human health raises important ethical, legal, and regulatory challenges. Gene editing of microbes, especially those introduced into human bodies, raises concerns about ecological disruption, unintended genetic consequences, and the potential for horizontal gene transfer. Furthermore, the long-term effects of genetically modified organisms on human health and the environment are not yet fully understood.

Research should prioritize the development of ethical frameworks and regulatory guidelines for the use of gene-edited microbes in clinical settings. This includes establishing safety standards, guidelines for long-term monitoring, and frameworks for ethical decision-making regarding gene-edited probiotics or therapies. Additionally, international cooperation on regulatory harmonization will be important to ensure that microbiome-based interventions are safe, effective, and sustainable across diverse populations.

### *In Vivo* Validation and Clinical Trials

Despite promising in vitro results, many microbiome-based therapies, particularly those involving AMPs or gene-edited probiotics, have yet to be extensively validated in human clinical trials. Moving from preclinical models to human trials is essential to determine the true therapeutic potential of these treatments.

There is a need for well-designed clinical trials that focus on the safety, efficacy, and long-term effects of microbiome-based interventions in humans. Specifically, randomized controlled trials should be conducted to assess the clinical outcomes of AMP therapies in infections, cancer, and inflammatory diseases. In parallel, real-world evidence from patients receiving these therapies will be crucial to understanding how individual variability in microbiomes affects treatment outcomes.

### Synergy Between CRISPR and AMPs: Clinical Efficacy and Applications

As the potential of both CRISPR gene editing technologies and AMPs expands, their combination offers a unique therapeutic advantage. CRISPR enables precise genetic modifications in microbes and pathogens, while AMPs provide direct antimicrobial effects. The integration of these two technologies holds significant promise in addressing the growing challenges of AMR and chronic infections. By combining CRISPR’s ability to engineer microbes and AMPs’ antimicrobial properties, clinicians could achieve enhanced therapeutic efficacy in several key ways.

#### CRISPR-Mediated Engineering of AMP-Producing Microbes

CRISPR can be used to genetically modify microbial strains, enabling them to produce AMPs more efficiently. This modification could lead to the development of probiotic strains capable of continuously synthesizing and releasing AMPs at therapeutic concentrations, providing sustained protection against infections. Engineered microbes could be introduced into patients, establishing long-term, low-maintenance antimicrobial defenses. This approach could prove invaluable in treating infections that are difficult to manage with conventional antibiotics.

#### Combating Pathogens with CRISPR and AMPs

The combined power of CRISPR and AMPs can be harnessed to target pathogens more precisely and effectively. CRISPR-based systems could be employed to modify pathogenic bacteria, such as knocking out resistance genes or altering their genetic makeup to make them more vulnerable to AMP action. Additionally, CRISPR could be used to develop bacteria or bacteriophages capable of specifically combating and neutralizing pathogenic strains, thus enhancing the potency of AMPs. This dual-combating approach not only amplifies antimicrobial activity but also reduces the likelihood of resistance development.

#### Overcoming Resistance Mechanisms

Resistance to both traditional antibiotics and AMPs is a growing concern, as pathogens evolve mechanisms to evade these treatments. CRISPR can be used to directly modify microbial resistance mechanisms, such as efflux pumps or protective biofilm formations, while AMPs continue to disrupt bacterial membranes or inhibit critical cellular functions. By attacking pathogens on two fronts, CRISPR and AMPs can work synergistically to combat resistant infections more effectively. This combination also provides a strategic advantage over traditional antibiotics, which may fail in the face of evolving resistance mechanisms.

#### Personalized Microbiome Therapies

Given the uniqueness of each individual’s microbiome, personalized approaches to therapy are crucial. CRISPR can be used to tailor treatments to an individual’s microbiome, potentially modifying the microbial community to produce specific AMPs suited to that person's health needs. By analyzing the metagenomic composition of a patient’s microbiome, CRISPR could enable the development of personalized probiotic therapies that not only target the patient’s microbial imbalances but also produce AMPs capable of addressing specific infections or disease states. This approach could enhance the specificity and efficacy of microbiome-based treatments, potentially reducing the need for broad-spectrum antibiotics and minimizing off-target effects.

#### Clinical Implications

The clinical integration of CRISPR and AMPs is an exciting prospect, offering the potential for more targeted, effective, and sustainable treatments. The combination of gene-edited microbes that produce AMPs and direct AMP therapies could revolutionize the treatment of infectious diseases, cancer, and even autoimmune disorders. However, the transition from preclinical research to clinical application will require rigorous testing, particularly in ensuring the long-term safety and efficacy of these treatments. Ethical considerations, regulatory challenges, and the potential ecological impact of gene-edited microbes will also need to be carefully addressed.

In clinical settings, the use of CRISPR-engineered microbes capable of producing AMPs could reduce the frequency of antibiotic use, lowering the risk of developing resistance. Personalized microbiome therapies could lead to more individualized treatments, improving outcomes and patient quality of life. As research progresses, clinical trials and real-world evidence will provide a clearer picture of how CRISPR and AMPs can complement each other in practice, opening the door to novel, more effective treatment paradigms.

## Challenges and Limitations

Despite the promise of microbiome-based therapies, particularly those involving AMPs and gene editing technologies, several practical challenges and limitations hinder their clinical translation. Addressing these challenges is essential for the successful application of these therapies.

### Regulatory and Ethical Constraints

CRISPR and AMP applications in clinical settings raise significant ethical concerns related to patient consent, equity, and long-term impacts. While personalized microbiome therapies hold great promise, they also present the challenge of ensuring equitable access, particularly to underserved populations. Informed consent is crucial, as patients must fully understand the potential risks, including unforeseen health consequences and long-term effects. Ethical considerations also extend to the possibility of unintended microbial evolution, which could have profound, long-lasting impacts on human health and the environment.

Additionally, the use of CRISPR-based gene editing technologies and engineered microbes in clinical settings faces considerable regulatory hurdles. Regulatory bodies must develop new guidelines to govern the use of genetically modified organisms in human microbiomes to ensure their safety and long-term effectiveness. Concerns about the unintended consequences of microbiome manipulation, such as environmental impact and the potential for horizontal gene transfer, remain significant barriers to the clinical application of these technologies. Ongoing research must address these ethical and regulatory challenges by developing comprehensive frameworks to guide the responsible use of CRISPR and AMP-based therapies.

To support the responsible development of CRISPR and AMP technologies, policymakers and clinicians should collaborate on the establishment of transparent, adaptable regulatory systems. These frameworks should be global in scope, enabling international harmonization of standards. Ethical frameworks should ensure ongoing evaluation of long-term effects and foster public engagement regarding the societal impacts of microbiome manipulation.

A comprehensive approach, which considers both innovation and public health protection, will be critical to the safe integration of CRISPR and AMP therapies into clinical practice.

### Resistance to AMPs

While AMPs hold great promise as antimicrobial agents, their susceptibility to microbial resistance presents a critical limitation. Just as bacteria have developed resistance to antibiotics, they can also evolve mechanisms to resist AMPs. Efflux pumps, membrane modifications, and protease degradation are all potential mechanisms that could compromise AMP efficacy over time. Addressing this issue requires developing AMP combinations or strategies to minimize resistance, but this remains a significant challenge in clinical application.

### Delivery and Bioavailability Challenges

The clinical use of AMPs is limited by their poor bioavailability and stability. AMPs are often rapidly degraded by proteolytic enzymes or eliminated from the body before they can exert their therapeutic effect, particularly in systemic applications. Furthermore, oral administration of AMPs is difficult due to their low absorption rates in the gastrointestinal tract. Overcoming these issues requires the development of advanced delivery systems, such as nanoparticle encapsulation, liposomal formulations, or peptide conjugation to carriers that protect AMPs and enhance their stability.

### Ecological Risks of Microbiome Manipulation

Microbiome interventions, whether through AMPs or gene editing, could unintentionally disrupt the natural balance of microbial ecosystems. The delicate balance of the microbiome can be easily disturbed, potentially leading to dysbiosis or the overgrowth of pathogenic species. Such disruptions could have unforeseen consequences on human health, leading to conditions such as IBD or metabolic disorders. Ensuring that microbiome manipulation does not disrupt microbial homeostasis is a major challenge that must be addressed in clinical applications.

### Clinical Validation and Safety Concerns

Despite promising preclinical data, the clinical validation of microbiome-based therapies remains a significant hurdle. Rigorous clinical trials are needed to assess the safety and efficacy of AMPs and gene-edited probiotics in humans. Challenges include variability in individual microbiomes, differences in patient responses to therapies, and long-term safety concerns. Clinical trials must address these complexities to ensure that microbiome-based therapies are both safe and effective for diverse patient populations.

### Complexity in Personalized Approaches

Tailoring microbiome-based therapies to individual patients presents challenges in understanding the unique microbial composition and functionality of each person’s microbiome. The variability in microbiome profiles complicates the design of universal therapies. Personalized treatment approaches will require integration of multi-omics data, such as metagenomics and metabolomics, to predict individual responses to therapies accurately. Developing reliable methods for personalized microbiome interventions will be a major challenge moving forward.

## Conclusions

In conclusion, microbiomes are vital to human health, influencing immune function, metabolism, and pathogen resistance. AMPs hold significant promise as alternatives to antibiotics and chemotherapy, particularly in combating multidrug-resistant infections and certain cancers. Advances in CRISPR-based gene editing have enabled precise microbial genome manipulation, opening new avenues for engineered probiotics and targeted therapies. Despite these advances, challenges remain, such as the ecological impact of microbiome manipulation, the bioavailability and stability of AMPs, and limitations in their clinical delivery. The next critical step is focused clinical research to validate these therapies, address long-term safety concerns, and explore their ecological consequences. Evolving regulatory frameworks will be crucial in ensuring the ethical and safe application of these therapies. Personalized medicine approaches will be key in tailoring treatments to individual microbiomes, further optimizing health outcomes. With continued research, microbiome-based therapies, especially those involving AMPs, hold the potential to revolutionize treatment strategies and improve overall health outcomes.

## Data Availability

No datasets were generated or analysed during the current study.

## Abbreviations

ACP: Anticancer peptide
AMP: Antimicrobial peptide
Cas9: CRISPR-associated protein 9
CRISPR: Clustered regularly interspaced short palindromic repeats
CRISPRi: CRISPR interference
dCas9: Dead Cas9 (mutated Cas9 without nuclease activity)
DSB: Double-strand break
FMT: Fecal microbiota transplantation
HDR: Homology-directed repair
HR: Homologous recombination
IBD: Inflammatory bowel disease
ICB: Immune checkpoint blockade
MIC50: Minimum inhibitory concentration required to inhibit 50% of microbial growth
MRSA: Methicillin-resistant *Staphylococcus aureus*
NHEJ: Non-homologous end joining
NK: Natural killer
PAM: Protospacer adjacent motif
PD-1: Programmed cell death protein-1
PTRK: Phage-derived tools for recombinase-mediated knockouts
SBVs: Synthetic bacterial vesicles
SCFA: Short-chain fatty acids
